# A New Score to Assess the Perioperative Period of the Cancer Patient Undergoing Non-Palliative Elective Surgery: A Retrospective Evaluation of a Case Report by PERIDIA Score

**DOI:** 10.3389/fonc.2021.733621

**Published:** 2021-10-11

**Authors:** Letizia Andresciani, Concetta Calabrò, Mariarita Laforgia, Maria Ronchi, Simona De Summa, Christel Cariddi, Rosa Boccuzzi, Anna De Rosa, Elisabetta Rizzo, Giulia Losito, Grazia Bradascio, Gaetano Napoli, Michele Simone, Giuseppe Carravetta, Giovanni Mastrandrea

**Affiliations:** ^1^ DETO Dipartimento di Emergenze e Trapianti d'Organo, Università degli Studi di Bari, Bari, Italy; ^2^ Unità Operativa Complessa Farmacia e UMACA, Istituto di Ricerca e Cura a Carattere Scientifico (IRCCS) Istituto Tumori Giovanni Paolo II–Bari, Bari, Italy; ^3^ Unità Operativa Complessa Chirurgia Generale Oncologica, Istituto di Ricerca e Cura a Carattere Scientifico (IRCCS) Istituto Tumori Giovanni Paolo II–Bari, Bari, Italy; ^4^ Diagnostica Molecolare e Farmacogenetica, Istituto di Ricerca e Cura a Carattere Scientifico (IRCCS) Istituto Tumori Giovanni Paolo II–Bari, Bari, Italy; ^5^ Unità Operativa Complessa Anestesia, Rianimazione e Terapia Intensiva PostOperatoria, Istituto di Ricerca e Cura a Carattere Scientifico (IRCCS) Istituto Tumori Giovanni Paolo II–Bari, Bari, Italy; ^6^ Unità Operativa Complessa Chirurgia Toracica, Istituto di Ricerca e Cura a Carattere Scientifico (IRCCS) Istituto Tumori Giovanni Paolo II–Bari, Bari, Italy

**Keywords:** perioperative score, peridiaphragmatic surgery, anesthesiology, ICU, cancer patients

## Abstract

The complexity of cancer patients and the use of advanced and demolitive surgical techniques frequently need post-operatory ICU hospitalization. To increase safety and to select the best medical strategies for the patient, a multidisciplinary team has performed a new peri-operatory assessment, arising from evidence-based literature data. Verifying that most of the cancer patients, admitted to the intensive care unit, undergo major surgery with localizations in the supramesocolic thoraco-abdominal area, the team focused the attention on supramesocolic peridiaphragmatic cancer surgery. Some scores already in use in clinical practice were selected for the peri-operatory evaluation process. None of them evaluate parameters relating to the entire peri-operative period. In detail, only a few study models were found that concern the assessment of the intra-operative period. Therefore, we wanted to see if using a mix of validated scores, it was possible to build a single evaluation score (named PERIDIAphragmatic surgery score or PERIDIA-score) for the entire peri-operative period that could be obtained at the end of the patient’s hospitalization period in post-operative ICU. The main property sought with the creation of the PERIDIA-score is the proportionality between the score and the incidence of injuries, deaths, and the length of stay in the ward. This property could organize a tailor-made therapeutic path for the patient based on pre-rehabilitation, physiotherapy, activation of social assistance services, targeted counseling, collaborations with the continuity of care network. Furthermore, if the pre-operative score is particularly high, it could suggest different or less invasive therapeutic options, and if the intra-operative score is particularly high, it could suggest a prolongation of hospitalization in ICU. The retrospective prospective study conducted on 83 patients is still ongoing. The first data would seem to prove an increase of clinical complications in patients who were assigned a one-third score with respect to the maximum (16/48) of PERIDIA-score. Moreover, patients with a 10/16 score within each phase of the evaluation (pre, peri, and post) more frequently develop injuries. In the light of these evidence, the 29-point score assigned to our patient can be considered as predictive for the subsequent critical and fatal complications the patient faced up.

## Introduction

The perioperative evaluation concerns the analysis of the characteristics of the cancer patient related to the possibility of undergoing surgery, the monitoring of vital functions in relation to surgical and anesthetic procedures during surgery, the primary and secondary prevention of complications related to surgery in post-operative intensive care unit.

The standardization of peri-operatory assessments in cancer patients undergoing peridiaphragmatic thoraco-abdominal surgery (such as esophagectomy, lobectomy and pneumonectomy, hepatic metastasectomy, pancreatectomy, gastrectomy, and splenectomy) is a very complex challenge, particularly in the case of multiorgan localization. This aim is worldwide pursued for each patient through the application of international evaluation scores in the pre-operatory step (fragility, nutritional structure, comorbidities, previous thoraco-abdominal problems) and/or the prediction of the post-operatory onset of complications.

To our knowledge, only a few experiences are reported in literature in terms of peri-operatory evaluation; in particular, the intra-operatory phase lacks shared and validated references as regards clinical scores in critical patients, passing through the three steps, pre-, intra-, and post-surgery.

The clinical data concerning 83 patients hospitalized in 2018 in post-operative ICU of the Cancer Institute Giovanni Paolo II of Bari were retrospectively analyzed. The following case report aims at demonstrating how a peri-operative evaluation is necessary to predict complications related to surgical treatment *versus* non-multidisciplinary and unstructured assessments. Our first results will be confirmed by an ongoing retrospective study on a large number of patients and by future prospective studies.

## Materials and Methods

**Figure 1:**
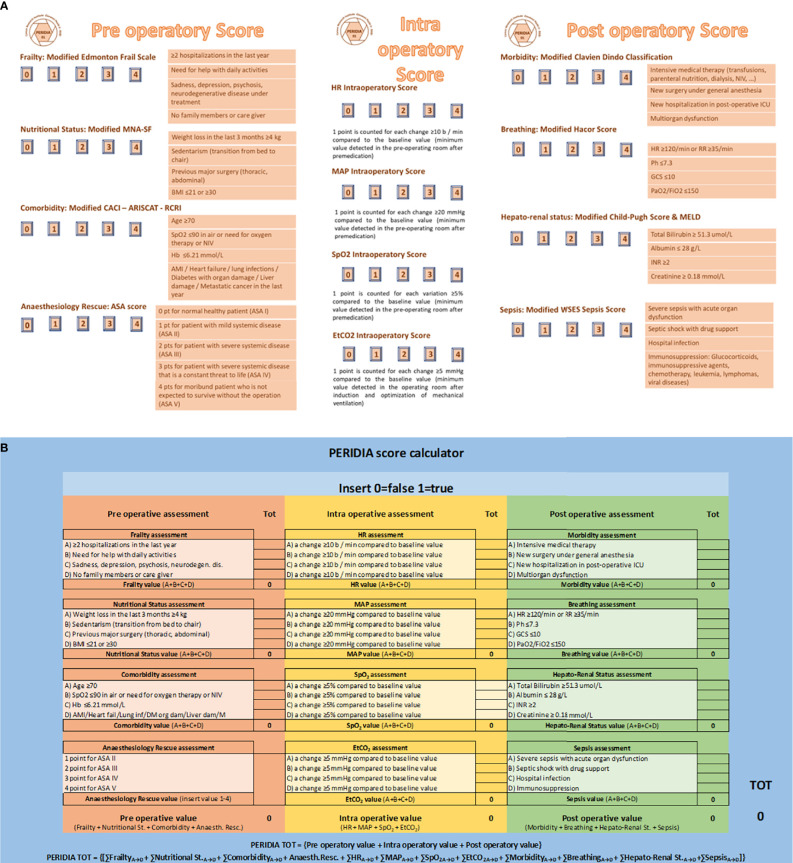
**(A)** PERIDIA SCORE, **(B)** PERIDIA Calculator. The PERIDIA score is divided into three scores (pre-operatory score, intra-operative score, post-operative score), each of which expressing a score from 0 to 16 for a maximum of 48 points. Pre-operatory score concerns the pre-operatory period and supports the anesthetic and surgical evaluations of pre-hospitalization. It consists of four scores, to each of which a result from 0 to 4 points can be assigned. Pre-operatory score estimates Frailty with the Modified Edmonton Frail Scale, Nutritional Status with the Modified Mini Nutritional Assessment Short Form, Comorbidity with the Modified Charlson Age Morbidity Index, ARISCAT Index, and Lee’s Revised cardiac Risk Index, Anaesthesiology Rescue with the ASA score. With regard to the Modified Edmonton Frail Scale, a point is assigned to the verification of each of the following conditions: ≥2 hospitalizations in the last year; need for help with daily activities; sadness, depression, psychosis, or neurodegenerative disease in treatment; absence of family members or caregivers. Regarding the Modified Mini Nutritional Assessment Short Form, a point is assigned to the verification of each of the following conditions: weight loss in the last 3 months ≥4 kg; sedentarism (transition from bed to chair); previous major surgery (thoracic, abdominal); BMI ≤21 or ≥30. About the Modified Charlson Age Morbidity Index, ARISCAT Index, and Lee’s Revised cardiac Risk Index, one point is assigned for each of the following conditions: age ≥70 years; SpO2 ≤90% in air environment or need for oxygen therapy or non-invasive ventilation; hemoglobinemia ≤6.21 mmol/L; AMI or heart failure or lung infections or diabetes with organ damage or liver injury or metastatic solid tumor in the last year. Finally, the ASA score is taken from the anesthetic assessment pre-hospitalization and in any case is assigned on the basis of the following criteria: 1 point for no organic, biochemical, or psychiatric alteration; 2 points for mild systemic disease related or not to the reason for the surgery; 3 points for severe but not disabling systemic pathology related or not to the reason for the surgery; 4 points for severe systemic disease with a severe prognosis that affects survival regardless of surgery. Intra-operative score assesses the variation of four vital parameters commonly used during the monitoring of general anesthesia (heart rate or HR, mean arterial pressure or MAP, saturation or SpO2, capnometry or EtCO2). So 1 point was assigned in the HR Intra-operatory Score for each change (±) ≥10 b/min with respect to the baseline value (minimum value detected after premedication); 1 point was assigned in the MAP Intra-operatory Score for each change (±) ≥20 mmHg with respect to the baseline value (minimum value detected after premedication); 1 point was assigned in the SpO2 Intra-operatory Score for each change ≥5% from the baseline value (minimum value detected after premedication), and 1 point was assigned in the EtCO2 Intra-operatory Score for each variation (±) ≥5 mmHg compared to the baseline value (minimum value detected after induction and optimization of mechanical ventilation). Post-operative score concerns the period of hospitalization in the ICU. It consists of four scores, to each of which a result from 0 to 4 points can be assigned. Post-operatory score estimates Morbidity with the Modified Clavien Dindo Classification, Breathing with the Modified Hacor Score, Hepato-Renal status with the Modified Child-Pugh Score & MELD, Sepsis with the Modified WSES Sepsis Score. Regarding the Modified Clavien Dindo Classification, a point was assigned to the occurrence of each of the following conditions: intensive medical therapy (transfusions, parenteral nutrition, dialysis, NIV, …); new surgical evaluation under general anesthesia; further hospitalization in post-operatory ICU; multiorgan dysfunction. About the Modified Hacor Score, a point was assigned to the occurrence of each of the following conditions: HR ≥120/min or RR ≥35/min; pH ≤7.3; GCS ≤10; PaO2/FiO2 ≤150. Regarding the Modified Child-Pugh Score & MELD, a point was assigned to the occurrence of each of the following conditions: Bilirubin tot ≥51.3 umol/L; Albumin ≤28 g/L; INR ≥2; Creatinine ≥0.18 mmol/L. With reference to the Modified WSES Sepsis Score, a point was assigned to the occurrence of each of the following conditions: severe sepsis with acute organ dysfunction; septic shock with vasopressors; nosocomial infection; immune suppression: glucocorticoids, immunosuppressive agents, chemotherapy, leukemia, lymphomas, viral diseases. ARISCAT Index, Assess Respiratory Risk in Surgical Patients in Catalogna; ASA score, American Society of Anesthesiologists score; BMI, Body Mass Index; EtCO2, End Tidal Carbon dioxide; GCS, Glasgow Coma Scale; HR, Heart Rate; INR, International Normalized Ratio; MAP, Mean Arterial Pressure; MELD, Model for End-Stage Liver Disease; NIV, Non-Invasive Ventilation; PERIDIA score, a new score used to the standardization of peri-operatory assessments in cancer patients undergoing peridiaphragmatic thoraco-abdominal surgery, particularly in the case of multiorgan localization; SPO2, Percentage Saturation of hemoglobin with Oxygen; WSES score, World Society of Emergency Surgery Sepsis score.

A multidisciplinary group, consisting of anesthesiologists, abdominal cancer surgeons and thoracic cancer surgeons, pharmacists, psychologists, statisticians, and nurses, has elaborated the PERIDIA Score (**Figures 1A, B**), starting from the analysis of the literature reference scores.

In the first step of our study, some scores already in use in clinical practice were selected for the peri-operatory evaluation process. Edmonton Frail Scale, Mini Nutritional Assessment Short Form, Charlson Age Morbidity Index, Assess Respiratory Risk in Surgical Patients in Catalogna (ARISCAT Index), Lee’s Revised Cardiac Risk Index, Preoperative Esophagectomy Risk, Clavien Dindo Classification, Child Pugh Score, Model for End Stage Liver Disease, Simple Risk Score for Pancreatectomy Surgical Outcomes Analysis and Research, Hacor Score, and World Society of Emergency Surgery Sepsis Score were deeply analyzed and synthetized by the team to extract the most significant items to build our new score, named PERIDIAphragmatic surgery score or PERIDIA score (1–20).

The PERIDIA score has been subsequently divided into three scores, each of which expressing a score from 0 to 16 for a maximum of 48 points. The first score concerns the pre-operatory period and supports the anesthetic and surgical evaluations of pre-hospitalization. It consists in four scores, to each of which a score from 0 to 4 points can be assigned. With regard to the Modified Edmonton Frail Scale, a point is assigned to the verification of each of the following conditions: ≥2 hospitalizations in the last year; need for help with daily activities; sadness, depression, psychosis, or neurodegenerative disease in treatment; absence of family members or caregivers. Regarding the Modified Mini Nutritional Assessment Short Form, a point is assigned to the verification of each of the following conditions: weight loss in the last 3 months ≥4 kg; sedentarism (transition from bed to chair); previous major surgery (thoracic, abdominal); BMI ≤21 or ≥30. About the Modified Charlson Age Morbidity Index, ARISCAT Index, and Lee’s Revised cardiac Risk Index, 1 point is assigned for each of the following conditions: age ≥70 years; SpO2 ≤90% in air environment or need for oxygen therapy or non-invasive ventilation; hemoglobinemia ≤6.21 mmol/L; AMI or heart failure or lung infections or diabetes with organ damage or liver injury or metastatic solid tumor in the last year. Finally, the ASA score is taken from the anesthetic assessment pre-hospitalization and in any case is assigned on the basis of the following criteria: 1 point for no organic, biochemical, or psychiatric alteration; 2 points for mild systemic disease related or not to the reason for the surgery; 3 points for severe but not disabling systemic pathology related or not to the reason for the surgery; 4 points for severe systemic disease with a severe prognosis that affects survival regardless of surgery.

For the intra-operative period, the multidisciplinary technical team aimed at matching the main phases of the surgery (T0: pre-treatment after pre-dressing; T1: post-induction; T2: post-cutting; T3: post-retractor or pneumo; T4: post-surgery; T5: post-anesthesia) with the variation of four vital parameters commonly used during the monitoring of general anesthesia (heart rate or HR, mean arterial pressure or MAP, saturation or SpO2, capnometry or EtCO2).

However, due to the lack of punctual clinical parameters in intra-operatory period, in the present case report, we had to adapt these evaluations, regardless of the surgical phase. Therefore, 1 point was assigned in the HR Intra-operatory Score for each change (±) ≥10 b/min with respect to the baseline value (minimum value detected after premedication); 1 point was assigned in the MAP Intra-operatory Score for each change (±) ≥20 mmHg with respect to the baseline value (minimum value detected after premedication); 1 point was assigned in the SpO2 Intra-operatory Score for each change ≥5% from the baseline value (minimum value detected after premedication), and 1 point was assigned in the EtCO2 Intra-operatory Score for each variation (±) ≥5 mmHg compared to the baseline value (minimum value detected after induction and optimization of mechanical ventilation).

The post-operative score is also based on four scores, each of which being assigned a score from 0 to 4 points. Regarding the Modified Clavien Dindo Classification, a point was assigned to the occurrence of each of the following conditions: intensive medical therapy (transfusions, parenteral nutrition, dialysis, NIV, …); new surgical evaluation under general anesthesia; further hospitalization in post-operatory ICU; multiorgan dysfunction. About the Modified Hacor Score, a point was assigned to the occurrence of each of the following conditions: HR ≥120/min or RR ≥35/min; pH ≤7.3; GCS ≤10; PaO2/FiO2 ≤150. Regarding the Modified Child-Pugh Score & MELD, a point was assigned to the occurrence of each of the following conditions: Bilirubin tot ≥51.3 umol/L; Albumin ≤28 g/L; INR ≥2; Creatinine ≥0.18 mmol/L. With reference to the Modified WSES Sepsis Score, a point was assigned to the occurrence of each of the following conditions: severe sepsis with acute organ dysfunction; septic shock with vasopressors; nosocomial infection; immune suppression: glucocorticoids, immunosuppressive agents, chemotherapy, leukemia, lymphomas, viral diseases.

## Results

**Figure 2:**
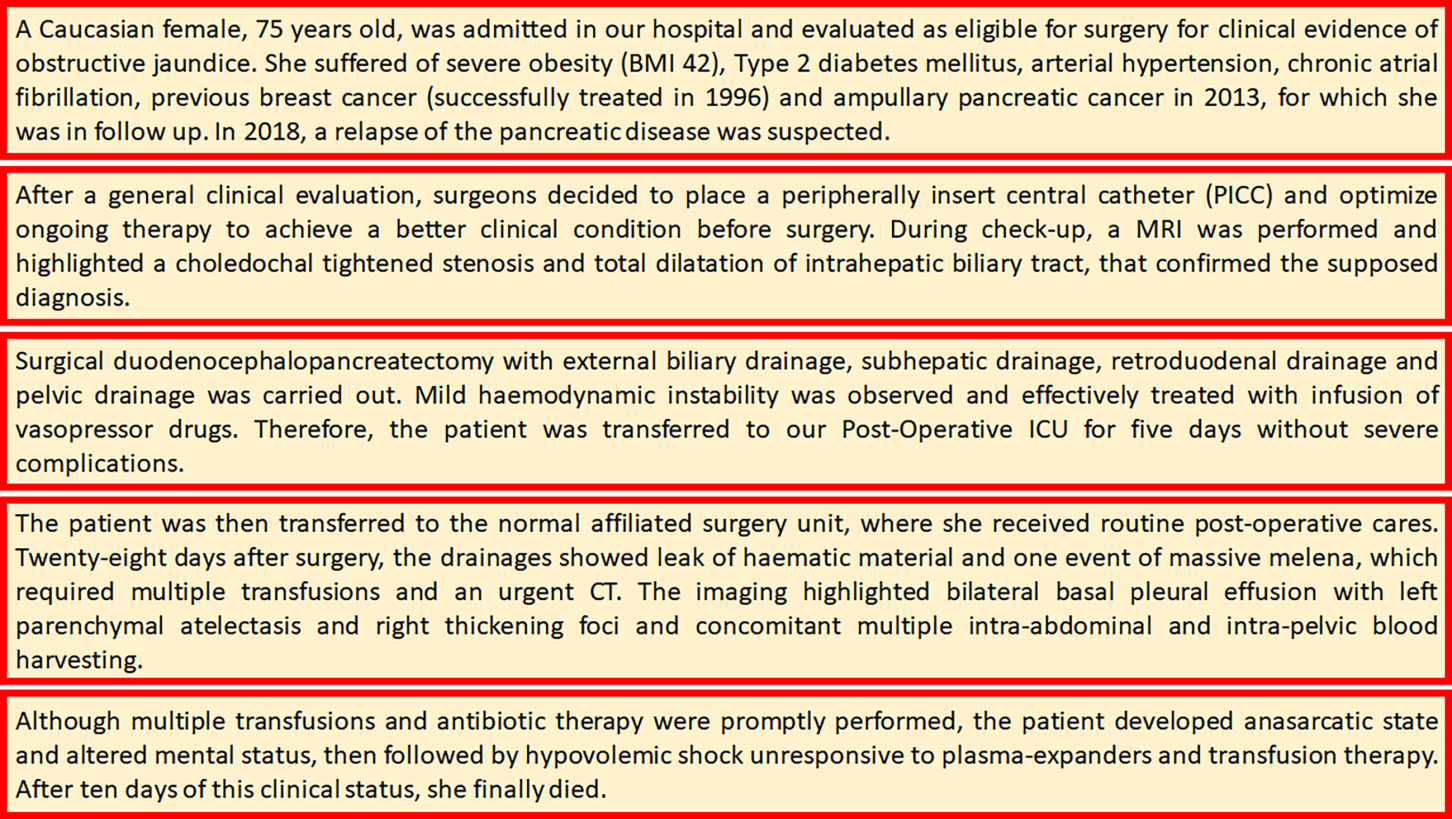
Case Presentation.

**Figure 3:**
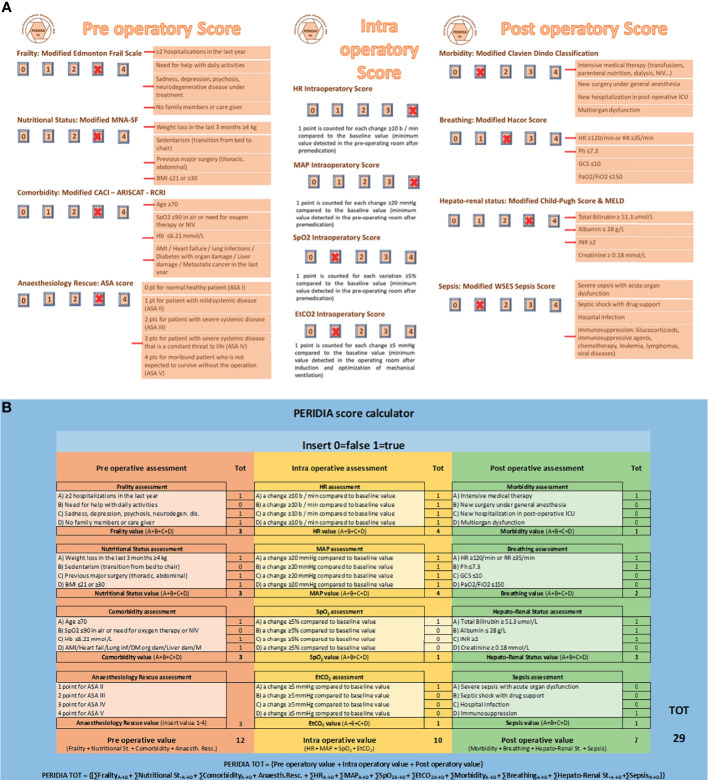
**(A)** PERIDIA SCORE applied to the patient, **(B)** PERIDIA Calculator applied to the patient. The score assigns 12/16 points in pre-operatory phase: 3 points for Frailty (1 point due to two hospitalizations in the last year, 1 point due to sadness and depression, 1 point due to sedentary lifestyle without caregivers), 3 points for Nutritional Status (1 point due to weight loss in the last 3 months of 8 kg, 1 point due to previous major abdominal surgery, 1 point due to BMI 42), 3 points for Comorbidity (1 point due to age 75 years, 1 point due to hemoglobinemia 5.28 mmol/L, 1 point due to diabetes mellitus with left lower limb neuropathy and local cancer recurrence), 3 points for Anesthesiology Rescue (anesthetic evaluation of ASA IV). In the intra-operatory period, the score assigned 10/16 points to the patient, due to four variations of HR ≥10 b/min, four variations of MAP≥20 mmHg, one variation of SpO2 ≥5%, one variation of EtCO2 ≥5 mmHg with respect to the baseline value, respectively. In the post-operatory period (ICU phase), the score assigned 7/16 points to the patient: 1 point for Morbidity (1 point due to the several transfusions and the necessity of parenteral nutrition), 2 points for Breathing (1 point due to HR 128/min, 1 point due to pH 7.25), 3 points for Hepato-Renal Status (1 point due to total Bilirubin 239.4 umol/L, 1 point due to Albumin 21 g/L, 1 point due to INR 2.5), 1 point for Sepsis (1 point due to previous chemotherapy). The X marks indicate the score awarded. The arrows indicate the parameter whose change caused the scoring. The total of score is 29/48 point. ARISCAT Index, Assess Respiratory Risk in Surgical Patients in Catalogna; ASA score, American Society of Anesthesiologists score; BMI, Body Mass Index; EtCO2, End Tidal Carbon dioxide; GCS, Glasgow Coma Scale; HR, Heart Rate; INR, International Normalized Ratio; MAP, Mean Arterial Pressure; MELD, Model for End-Stage Liver Disease; NIV, Non-Invasive Ventilation; PERIDIA score, a new score used to the standardization of peri operatory assessments in cancer patients undergoing peridiaphragmatic thoraco-abdominal surgery, particularly in the case of multiorgan localization; SPO2, Percentage Saturation of hemoglobin with Oxygen; WSES score, World Society of Emergency Surgery Sepsis score.

The PERIDIA score was applied to the patient (**Figures 2**, **3A, B**). In the pre-operatory period, the score assigned 12 points to the patient due to two hospitalizations in the last year, sadness and depression, sedentary lifestyle without caregivers, weight loss in the last 3 months of 8 kg, previous major abdominal surgery, BMI 42, age 75 years, hemoglobinemia 5.28 mmol/L, diabetes mellitus with left lower limb neuropathy and local cancer recurrence, anesthetic evaluation of ASA IV.

In the intra-operatory period, the score assigned 10 points to the patient, due to four variations of HR ≥10 b/min, four variations of MAP ≥20 mmHg, one variation of SpO2 ≥5%, one variation of EtCO2 ≥5 mmHg with respect to the baseline value, respectively.

In the post-operatory period, the score assigned 7 points to the patient due to the several transfusions and the necessity of parenteral nutrition, HR 128/min, pH 7.25, total Bilirubin 239.4 umol/L, Albumin 21 g/L, INR 2.5, previous chemotherapy.

The total PERIDIA score was 30 points. Due to the numerous adhesions related to the previous surgical procedure, the last surgery lasted 8 h; in post-operative ICU the patient stayed 5 days, while the whole hospitalization was 61 days.

## Discussion

After a previous cancer, the patient was affected by a relapse of a pancreatic tumor, with a poor prognosis, with local recurrence in a context of comorbidities (arrhythmia, jaundice, metabolic syndrome) that was presumably not adequately assessed for the possibility/need for surgery.

In these clinical cases, a peri-operative evaluation score able to trace the right route of treatment could provide alerts both in the pre-operative period (for example, the possibility to start a tailored prehabilitation path or a surgical procedure rather than a path of palliative care) and in the intra-operative (need to use invasive monitoring of cardiac output by catecholamines in continuous infusion) and in the post-operative, for instance for a prolonged hospitalization, the destination of a semi-intensive post-operative room, where the patient can receive a continuous monitoring of vital functions.

The multidisciplinary team assigned a 29/48 score to the patient. This value is far beyond the upper threshold we are defining as a minimum score to identify possible predictable risks, according to our first results in the ongoing retrospective PERIDIA01 study. This study is demonstrating an increase of clinical complications in patients who were assigned a one-third score with respect to the maximum (16/48). Moreover, patients with a 10/16 score within each phase of the evaluation (pre, peri, and post) more frequently develop clinical complications.

In the light of these evidence, the 29-point score assigned to our patient can be considered as predictive for the subsequent critical and fatal complications the woman faced up.

The use of a peri-operative score elaborated by a multidisciplinary team even if in retrospective evaluation also allows to formulate other considerations on the clinical course of the patient, in particular from a pharmacological point of view. The patient administered before and during hospitalization Diltiazem Hydrochloride 60 mg (1/2 tablet twice a day), Digoxin 0.125 mg (one tablet a day), Warfarin (one tablet a day), Irbersartan + Hydrochlorothiazide 150 mg +12.5 mg (one tablet a day). At the hospitalization, she showed significant extension of the INR and electrolyte alteration.

This clinical condition could derive also from pharmacological interactions. In fact, Hydrochlorothiazide can produce hypokalemia and hypomagnesemia, which increase the inhibition of Na/K ATPasi mediated by Digoxin. Furthermore, Diltiazem may cause increases in digoxin plasma levels, probably by decreasing digoxin clearance. Hypokalemia and hypomagnesemia induced by diuretics may predispose patients on digitalis treatment to arrhythmias.

During the 2 months of hospitalization, the patient received Furosemide 20 mg (twice a day). The combination with a thiazide loop diuretic drug (Hydrochlorothiazide) may produce additive or synergistic effects on diuresis and excretion of electrolytes including sodium, potassium, magnesium, and chloride. This condition could explain the electrolytic alteration. Furthermore, the patient was treated with proton pump inhibitor Pantoprazole 40 mg per day, which is reported to induce hypomagnesemia in chronic use, and the risk may be further increased when combined with other medications such as furosemide. Although diuretics and digitalis glycosides are frequently and appropriately used together, diuretic-induced hypokalemia and hypomagnesemia may predispose these patients on digitalis treatment to arrhythmias. In fact, during hospitalization, cardiologists reported arrhythmic tones.

In the light of the results obtained from the application of the PERIDIA score in some patients, our multidisciplinary team intends to continue the application of this new evaluation system retrospectively to a larger cohort of patients to provide a further scaling up in the assessment process of the peri-operative score in oncologic patients, with a particular reference also to the pharmacologic treatment to choose in each step of the care pathway (22–27).

## Data Availability Statement

The original contributions presented in the study are included in the article/**Supplementary Material**. Further inquiries can be directed to the corresponding author.

## Ethics Statement

The studies involving human participants were reviewed and approved by Comitato Etico–IRCCS Istituto Tumori “Giovanni Paolo II”–Bari, Italy. The patients/participants provided their written informed consent to participate in this study. Written informed consent was obtained from the individual(s) for the publication of any potentially identifiable images or data included in this article.

## Author Contributions

All authors listed have made a substantial, direct, and intellectual contribution to the work and approved it for publication.

## Funding

Support was provided solely from institutional and/or departmental sources.

## Conflict of Interest

The authors declare that the research was conducted in the absence of any commercial or financial relationships that could be construed as a potential conflict of interest.

## Publisher’s Note

All claims expressed in this article are solely those of the authors and do not necessarily represent those of their affiliated organizations, or those of the publisher, the editors and the reviewers. Any product that may be evaluated in this article, or claim that may be made by its manufacturer, is not guaranteed or endorsed by the publisher.
